# Effects of a Newly Developed Enzyme-Assisted Extraction Method on the Biological Activities of Fucoidans in Ocular Cells

**DOI:** 10.3390/md18060282

**Published:** 2020-05-26

**Authors:** Philipp Dörschmann, Maria Dalgaard Mikkelsen, Thuan Nguyen Thi, Johann Roider, Anne S. Meyer, Alexa Klettner

**Affiliations:** 1 Department of Ophthalmology, University Medical Center, University of Kiel, Arnold-Heller-Str. 3, Haus 25, 24105 Kiel, Germany; johann.roider@uksh.de (J.R.); alexakarina.klettner@uksh.de (A.K.); 2 Department of Biotechnology and Biomedicine, Technical University of Denmark, Søltofts Plads, 2800 Kongens Lyngby, Denmark; mdami@dtu.dk (M.D.M.); thuthi@dtu.dk (T.N.T.); asme@dtu.dk (A.S.M.)

**Keywords:** fucoidan, fucose, enzymatic purification, age-related macular degeneration, VEGF, oxidative stress, *Laminaria digitata*, *Fucus distichus* subsp. *evanescens*, *Saccharina latissima*, retinal pigment epithelium

## Abstract

Fucoidans from brown seaweeds are promising substances as potential drugs against age-related macular degeneration (AMD). The heterogeneity of fucoidans requires intensive research in order to find suitable species and extraction methods. Ten different fucoidan samples extracted enzymatically from *Laminaria digitata* (LD), *Saccharina latissima* (SL) and *Fucus distichus* subsp. *evanescens* (FE) were tested for toxicity, oxidative stress protection and VEGF (vascular endothelial growth factor) inhibition. For this study crude fucoidans were extracted from seaweeds using different enzymes and SL fucoidans were further separated into three fractions (SL_F1-F3) by ion-exchange chromatography (IEX). Fucoidan composition was analyzed by high performance anion exchange chromatography (HPAEC) after acid hydrolysis. The crude extracts contained alginate, while two of the fractionated SL fucoidans SL_F2 and SL_F3 were highly pure. Cell viability was assessed with an 3-(4,5-dimethylthiazol-2-yl)-5-(3-carboxymethoxyphenyl)-2-(4-sulfophenyl)-2H-tetrazolium (MTS) assay in OMM-1 and ARPE-19. Protective effects were investigated after 24 h of stress insult in OMM-1 and ARPE-19. Secreted VEGF was analyzed via ELISA (enzyme-linked immunosorbent assay) in ARPE-19 cells. Fucoidans showed no toxic effects. In OMM-1 SL_F2 and several FE fucoidans were protective. LD_SiAT2 (Cellic^®^CTec2 + Sigma-Aldrich alginate lyase), FE_SiAT3 (Cellic^®^ CTec3 + Sigma-Aldrich alginate lyase), SL_F2 and SL_F3 inhibited VEGF with the latter two as the most effective. We could show that enzyme treated fucoidans in general and the fractionated SL fucoidans SL_F2 and SL_F3 are very promising for beneficial AMD relevant biological activities.

## 1. Introduction

AMD (age-related macular degeneration) as main cause of central vision loss in the elderly is an irreversible disease with the number of patients annually increasing [1]. In the late phase of the disease, two forms exist which both lead to a degeneration of retinal components in the macula lutea. In the early form of AMD oxidized lipid protein molecules are deposited, terminating in accumulated drusen, which may interfere with retinal pigment epithelium (RPE) function. RPE cells are important for the maintenance of the photoreceptors. In the late stages of the disease geographic atrophies can occur, with large areas of RPE and photoreceptor degeneration [2,3]. In the exudative (“wet”) late form, an excessive production of the vascular endothelial growth factor (VEGF) leads to the formation of new blood vessels growing under and into the retina, causing edema and bleeding which ruptures the retina [2]. The pathology and causes of AMD development are not completely understood, but factors like complement activation, oxidative stress, inflammatory milieu and the excess VEGF are correlated with the development of AMD [2,4,5,6]. Up until now, the only treatment available are VEGF inhibiting agents, which need to be regularly injected in the human eye and slow down the deterioration but cannot cure the disease [7].

Brown seaweeds produce numerous very promising chemical compounds that are interesting for medical research, because of their beneficial effects for human health. An example are fucoidans, also designated as sulfated fucans, which are marine polysaccharides mainly composed of sulfated fucosyl moieties and sulfate ester groups. Minor constituents in fucoidans include other sugar moieties like galactose, mannose, xylose and glucuronic acids. Fucoidans are cell wall components and serve mainly as protective agent against pathogens and other environmental influences in the ocean [8]. In addition, they are also important as structural component and protect against dehydration [9]. Fucoidans exert many additional biological effects. These biological activities depend on the structure and this in turn depends on factors like algal species [10], harvest place and harvest time [11]. Among the different biological effects are the capability to lower inflammatory cytokines, to reduce oxidative burden and to inhibit VEGF as well as blood lipids [12]. These effects pave the way for a possible treatment option for AMD and other diseases in the human eye [13]. However, the activities of the fucoidans are highly dependent on the biological systems they are applied to. Therefore, appropriate testing systems are vital for investigating its potential and furthermore, the extraction method, as this effects the structure and the purity of the tested extracts are of high importance for reproducible beneficial effects.

In order to properly elucidate structure-function relationships of fucoidans for prevention of AMD or other degenerative diseases, it is essential to focus on the extraction technology. In particular, to obtain pure fucoidans, while maintaining the relevant structural features required for specific biological activities. Early work on fucoidans relied on several steps of acidic extraction at elevated temperatures (70–100 °C), but such extractions may affect the chemical composition and size of the extracted fucoidans [14]. Instead, we have developed several targeted enzymatic assisted extraction procedures that gently and precisely loosens up the cell wall matrix, releasing fucoidans in a gentle way, obtaining crude fucoidan extracts, containing also low molecular weight alginates In previous work one of these new enzymatic treatments were used and followed by ion-exchange chromatographic (IEX) purification obtaining well-defined, pure fucoidans from *Saccharina latissima* (SL) [15].

We showed already promising effects of different fucoidans on ocular cells. In brief, fucoidans from past studies were extracted with hot water, followed by precipitation with CaCl_2_ and ultrafiltration or dialysis [16,17,18,19]. *Fucus vesiculosus* fucoidan from Sigma-Aldrich can reduce angiogenesis and VEGF of RPE [13]. Fucoidans from *Fucus serratus*, *Laminaria digitata* (LD) and *Fucus distichus* subsp. *evanescens* (FE) were protective against oxidative stress in the uveal melanoma cell line OMM-1 and could inhibit ARPE-19 VEGF production [19] exactly like a other *Saccharina latissima* fucoidan, which was protective in ARPE-19 and lowered VEGF of primary RPE [19]. Different sized fucoidans from *Laminaria hyperborea* showed that the large, non-degraded fucoidan is most suitable for oxidative stress protection and VEGF inhibition [17]. Moreover, the tested fucoidans are not antiproliferative for ocular cells in general [16], which is necessary for use in medical treatments. However, the biological effects of fucoidan differ strongly in relation to their chemical characteristics, which are influenced by the extraction method, and activities may be confounded by contaminants in the extracts [20,21].

The objective of this work was to examine fucoidans from three different algal species (LD, SL, FE), which were extracted with four different enzymatic treatments, followed by alginate precipitation with either HCl or CaCl_2_. Additionally, crude fucoidan from SL was further purified and separated by ion-exchange chromatographic (IEX). In our previous studies related to fucoidans and their effect on ocular cells, we focused on the comparison of different algal species, fucoidans with different molecular weights or the effects on cell viability in tumor and non-tumor cell lines. In this study we tested fucoidans from different species, extracted from the seaweeds by different enzymatic methods. In addition, SL extracts were further purified and fractionated by IEX thereby removing contaminating compounds like alginate and polyphenols and achieving a higher fucose content. This study focuses on investigating whether the biological activity of fucoidans can be improved by different enzyme assisted purification methods. We compare fucoidans from different brown algal species and different enzymatically assisted treatments as well as IEX fractionation, to choose the most promising combination for further AMD research. Performed tests include detection of toxic effects, the ability to protect ocular cells against oxidative stress and to inhibit VEGF secretion. Furthermore, molecular weights and monosaccharide composition was determined to make a connection to the biological effects. Taken together, this study is well equipped to compare the bioactivity of fucoidans in relation to enzymatic extraction methods (different FE enzymatic purified extracts), further isolating steps (fractionated SL extracts), and also different species of origin (LD, SL, FE) in relation to the molecular weight and monosaccharide composition.

## 2. Results

### 2.1. Chemical Characterization of the Fucoidans

All tested fucoidans and the used extraction and purification methods were designated according to a code as seen in Table 1 In brief, the dried seaweed material was enzymatically extracted with commercial cellulase preparations Cellic^®^CTec2 or Cellic^®^CTec3 mixes (“2” or “3” in extraction code) and additional alginate lyase from Sigma-Aldrich SigmALy (SiAT) or alginate lyase SALy expressed from *Sphingomonas* sp. (SAT) were added (making the extracts SiAT2, SiAT3 as well as SAT2 and SAT3). The extracts with an additional “ad” in the code were precipitated with acid (HCl). Other extracts were precipitated with CaCl_2_. F1–F3 stands for an additional three-step fractionation process with chromatographic isolation (IEX).

Three crude extracts were made with Cellic^®^CTec2 and SigmALy from the three different algae (LD_SiAT2, SL_SiAT2 and FE_SiAT2). The residual alginate was not precipitated in these extracts and the alginate content was high (mannuronic acid plus guluronic acid) 87.2%, 80.3% and 67.5% and the fucose content was low 3.9, 12.3 and 15.5% respectively, as determined by high performance anion exchange chromatography (HPAEC) with pulsed amperometric detection (PAD) (Table 2; [22]) (chemical composition with standard deviations in Table A1).

In order to see the highest values of each substance instantly, we marked these values in bold. Not all alginate was degraded by the alginate lyase SigmALy, therefore the following extracts were prepared with different enzyme mixes and with further alginate precipitation with acid followed by neutralization of pH and dialysis (FE_SiAT2ad, FE_SiAT3ad, FE_SAT2ad, FE_SAT3ad). Since it contains the highest amount of fucoidan FE was used for these extractions. The resulting extracts had a comparable lower content of alginates 37.1, 50.4, 21.3 and 28.0% and higher content of fucose 36.1, 35.9, 52.2, 48.3%, respectively, compared to the previous extract FE_SiAT2. Furthermore, it was evident that the use of the alginate lyase SALy was more efficient in degrading the alginate than SigmALy, in particular the mannuronic acids (Table 2). These optimizations of using SALy and alginate precipitation was used to prepare highly pure and fractionated fucoidans from *S. latissima* by ion-exchange chromatography (IEX), the purification method was described previously [15], while the bioactivity is tested here. SL was chosen, since previous work has shown that SL fucoidan has biological effects [19]. A further optimization was used, were the alginate was precipitated by CaCl_2_ instead of acid, this method is believed to be gentler and is therefore likely to preserve the fucoidan structure better than the use of acid. Crude extracts from SL and FE using this method contained comparable total fucose yields and sulfate content, compared to a mild chemical extraction using acid [15]. In comparison to the LD extract three fractions of fucoidans were obtained by IEX, SL_F1, SL_F2 and SL_F3. The first elution SL_F1 contained almost exclusively alginates (90.9%) of lower molecular weight (4 kDa; [15]) while the fucose content was 5%. The SL_F2 and SL_F3 extracts contained very low amounts of alginates (6.9% and 0.8%) and high fucose content (64.7 and 63.3%), respectively. Furthermore, the SL_F2 and SL_F3 extracts contained a considerable amount of galactose, which is a likely constituent of SL fucoidan (12.2 and 26.9%) [15]. 

The sulfate content was previously determined for the SL fractions and corresponds well to the high amount of fucoidan present in the F2 and F3 fractions. The sulfate content for SL_F1, F2 and F3 was 6.6 ± 3.6, 35.6 ± 2.5 and 46.4 ± 3.5% respectively [15].

The size of the fucoidans was determined using high performance size-exclusion chromatography (HP-SEC). The calculated size of the fucoidans was between ~250 to over 800 kDa, with a general broad estimated size distribution (Table 3). Fucoidans from FE were generally around 350 kDa with an estimated distribution from 200–500 kDa, while the crude extracts of SL and LD were smaller, with a size around 250 and 320 kDa, respectively. The SL_F1 fraction contained mostly low molecular weight alginates with a size around 10 kDa, a peak which was also present in all crude extracts. The comparably low amount of fucoidans made it hard to determine the size in the SL_F1 extract. The size distribution was comparable and very large for SL_F2 and F3, and ranged from 100–1000 kDa, with a calculated size of over 800 kDa.

### 2.2. Effects on Cell Viability

**Figure 1:**
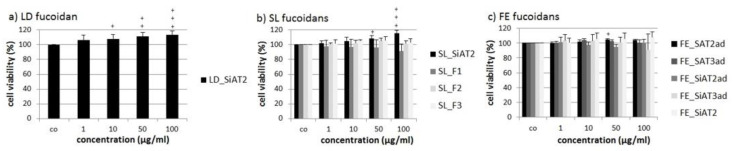
The cell viability of the uveal melanoma cell line OMM-1 was assessed after treatment for 24 h with *Laminaria digitata* (LD) fucoidan (**a**), *Saccharina latissima* (SL) fucoidans (**b**) and *Fucus distichus* subsp. *evanescens* (FE) fucoidans (**c**) extracted with SiAT2/3 or SAT2/3 (SiAT2/3 = Cellic^®^CTec2 or 3 enzyme mix + Sigma-Aldrich alginate lyase (SigmALy), SAT2/3 = Cellic^®^CTec2 or 3 enzyme mix + alginate lyase expressed from *Sphingomonas* sp. (SALy), ad = acid treatment and dialysis). Also, three SL ion-exchange chromatography (IEX) fractions (SL_F1, SL_F2 and SL_F3) were invastigated. Cell viability was analyzed with a MTS (3-(4,5-Dimethylthiazol-2-yl)-5-(3-carboxymethoxyphenyl)-2-(4-sulfophenyl)-2H-tetrazolium) assay and is shown as the mean and standard deviation in relation to the 100% control. Significance was determined with ANOVA; + *p* < 0.05, ++ *p* < 0.01, +++ *p* < 0.001 compared to control (*n* ≥ 4; number of independent experiments). No fucoidan exhibited antiproliferative effects.

Cell viability of the uveal melanoma cell line OMM-1 was determined after treatment with the different fucoidans for 24 h using the commercially available 3-(4,5-dimethylthiazol-2-yl)-5-(3-carboxymethoxyphenyl)-2-(4-sulfophenyl)-2H-tetrazolium (MTS) assay (Figure 1). None of the tested fucoidans significantly lowered cell viability, but some significantly increased the viability, for instance 100 µg/mL LD_SiAT2 increased viability up to 113% ± 6% (*p* < 0.001) and the SL fucoidan SL_SiAT2 increased viability to 115% ± 4% (*p* < 0.001). This might be related to the SiAT2 extraction method (in case of SL and LD fucoidan), which perhaps leads to fucoidans with beneficial effects for the cell viability of OMM-1 cells.

**Figure 2:**
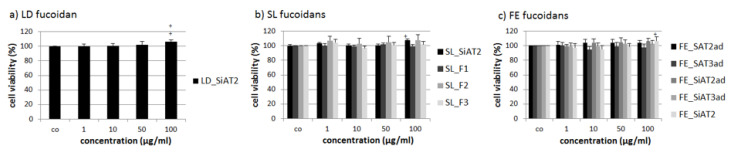
The cell viability of the human RPE cell line ARPE-19 was assessed after treatment for 24 h with LD fucoidan (**a**), SL fucoidans (**b**) and FE fucoidans (**c**). Cell viability was analyzed with a MTS assay and is shown as the mean and standard deviation in relation to the 100% control. Significance was determined with ANOVA; + *p* < 0.05, ++ *p* < 0.01 compared to control (*n* ≥ 4, number of independent experiments). No fucoidan showed antiproliferative effects.

The same procedure was performed for the human RPE cell line ARPE-19 (Figure 2). Cell viability was determined after 24 h with the MTS assay. Again, none of the fucoidan displayed significant antiproliferative effects. SiAT2 extracts from all three seaweed species, increased cell viability slightly at 100 µg/mL, again, although under 10% difference compared to control and therefore not biological relevant.

### 2.3. Effects on Oxidative Stress Protection

**Figure 3:**
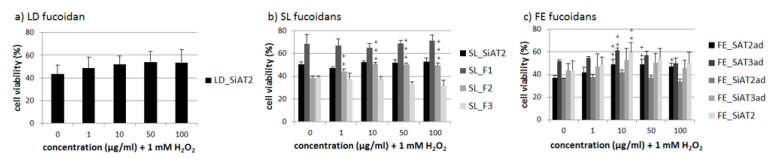
OMM-1 cell survival after 30 min treatment with LD fucoidan (**a**), SL fucoidans (**b**) and FE fucoidans (**c**) and 24 h stress insult with 1 mM H_2_O_2_, which reduced cell viability to at least 60% in all cases. Viability was determined with MTS assay. Values are pictured as the mean and standard deviation in relation to an untreated control (100%). Significance was evaluated via ANOVA; + *p* < 0.05, ++ *p* < 0.01, +++ *p* < 0.001 versus 0 µg/mL fucoidan + 1 mM H_2_O_2_ (*n* ≥ 4, number of independent experiments).

The LD fucoidan showed no significant protective effects against any tested concentration of H_2_O_2_, in the melanoma cell line OMM-1 (Figure 3). From SL, only the SL_F2 increased cell viability significantly at all concentrations tested with 10 µg/mL and 50 µg/mL showing the best protection (both 51% ± 1, *p* < 0.001 against 39% ± 1 stress control). FE fucoidans showed heterogeneous results depending on the tested concentration and used extraction method. FE_SAT2ad showed significant protective effects (49% ± 4%, *p* < 0.01; 49% ± 5%, *p* < 0.01; 47% ± 3%, *p* < 0.05 against 37% ± 2% stress control) at different concentrations 10 µg/mL, 50 µg/mL and 100 µg/mL, respectively In addition,10 µg/mL FE_SAT3ad and FE_SiAT2 increased cell viability up to 61% ± 2%, *p* < 0.01 and 60% ± 8%, *p* < 0.01. It seems that the protective effects are more dependent on the tested FE fucoidan concentration then on the extraction method.

**Figure 4:**
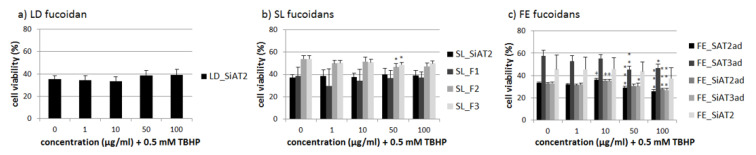
ARPE-19 cell survival after 30 min treatment with LD fucoidan (**a**), SL fucoidans (**b**) and FE fucoidans (**c**) and 24 h stress insult with 0.5 mM TBHP *(tert*-butyl hydroperoxide), which reduced cell viability below 60% in all cases. Viability was determined with MTS assay. Values are pictured as the mean and standard deviation in relation to an untreated control (100%). Significance was evaluated via ANOVA; + / * *p* < 0.05, ** *p* < 0.01, *** *p* < 0.001 versus 0 µg/mL fucoidan + 0.5 mM TBHP (*n* ≥ 4, number of independent experiments).

One role of RPE cells is to limit the oxidative stress in the human retina [4]. ARPE-19 cells as an RPE cell line are very resistant against hydrogen peroxide [26]. Therefore we used 0.5 mM *tert*-butyl hydroperoxide (TBHP) to lower the cell viability of ARPE-19 significantly after 24 h, as previously shown [19]. Again, the LD fucoidan showed no significant effect (Figure 4). Some of the FE fucoidans also had a slight additional toxic effect at 50 and 100 µg/mL, while 10 µg/mL FE_SAT2ad, FE_SiAT2ad and FE_SiAT3ad had a minimal protective effect. FE fucoidan at 10 µg/mL seems to be the best concentration concerning oxidative stress protection, but the effects are small and not relevant, corresponding to the fact that ARPE-19 are rather resistant against oxidative stress on their own and are hardly affected by fucoidan extracts [17,19]. SL_F2 and SL_F3 at concentrations of 50 µg/mL slightly decreased cell viability significantly down to 47% ± 3, *p* < 0.05 and 49% ± 2%, *p* < 0.05, respectively compared to 54% ± 3% stress control, but this not likely to be biological relevant. Otherwise, there were no significant effects for the SL fucoidans. 

### 2.4. VEGF Secretion of ARPE-19

We tested the influence of the ten different fucoidans on the VEGF secretion of the human RPE cell line ARPE-19. The optimal parameters for VEGF determination after fucoidan treatment were determined in a previous study [11]. In brief, cells were incubated for three days with the fucoidans and media exchange was done 24 h before taking of the supernatant for a subsequent ELISA analysis. VEGF in % was set in relation to cell viability in % both compared to untreated control (in arbitrary units [arb. unit]. The cell viability of both cell types was essentially unaffected by treatment with any sulfated fucans and at any tested concentrations (data not shown).

**Figure 5:**
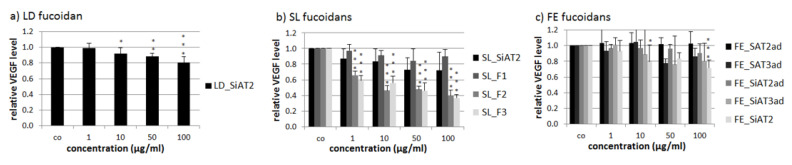
Secreted VEGF (vascular endothelial growth factor) of ARPE-19 after three days of incubation with 1, 10, 50 and 100 µg/mL LD fucoidan (**a**), SL fucoidans (**b**) and FE fucoidans (**c**). VEGF amount was determined with ELISA and normalized to cell survival, making a quotient of VEGF and cell viability. 10–100 µg/mL LD_SiAT2 and 1–100 µg/mL SL_F2 and SL_F3 decreased VEGF significantly. Significant values were analyzed with ANOVA, * *p* < 0.05, ** *p* < 0.01, *** *p* < 0.001 compared to the control (*n* ≥ 4, number of independent experiments).

LD_SiAT2 lowered secreted VEGF at 10, 50 and 100 µg/mL to 0.92 ± 0.08 [arb. unit (arbitrary unit)] (*p* < 0.05), 0.88 ± 0.04 [arb. unit] (*p* < 0.01) and 0.81 ± 0.07 [arb. unit] (*p* < 0.001), respectively (Figure 5). SL_F2 and SL_F3 reduced VEGF significantly at all tested concentration, in contrast to the first fraction SL_F1 and the unfractionated SL_SiAT2 with the highest effect at 100 µg/mL (SL_F2 with 0.40 ± 0.07 [arb. unit], *p* < 0.001 and SL_F3 with 0.37 ± 0.04 [arb. unit], *p* < 0.001). The FE extracts did not show any significant VEGF reducing effects, which could be also due to the high standard deviation and heterogeneous results, with the only exception of FE_SiAT2, which lowered VEGF significant at 10 µg/mL to 0.80 ± 0.21 [arb. unit], *p* < 0.05 and at 100 µg/mL to 0.72 ± 0.10 [arb. unit], *p* < 0.001.

## 3. Discussion

### 3.1. Integration in Previous Studies

This study is the continuation of fucoidan research within the EU FucoSan project in regards to finding a possible treatment for AMD [16,17,18,19]. One main goal of this international project is to characterize different fucoidans to choose the best fucoidan for a potential medical application. Factors like algae species, extraction method, purity, chemical composition, harvest place and time are important to define fucoidans with the best beneficial effects for further treatment development in AMD. In previous studies, factors like algae species and molecular weight were in focus. We could show that fucoidans from SL and LH (extracted with hot water extraction followed by CaCl_2_ precipitation and ultrafiltration or dialysis) showed the most promising effects of the species tested so far. In addition, high-molecular weight correlates with beneficial activities relevant for AMD [17,19]. Another study, testing fucoidan of FE, gave first indication that purity is an important factor for the relevant biological activities [18]. It has been suspected that in addition to the algae species the extraction method is a huge influencing factor concerning the biological activity, because it influences the structure and overall composition of the extract, leading to differential effects dependent on the method of extraction. Therefore, we investigated fucoidans purified by a novel technique using different enzymes. Additionally, we compared the effects in the three species LD, SL and FE, with different molecular weights and different monosaccharide compositions, to elucidate the best suited extraction method and algal species.

### 3.2. Slightly Increased Cell Viability in OMM-1 and ARPE-19 Relation to Uronic Acids, Molecular Weight and Concentration

Toxicity was not found after treatment with any extracts, which corresponds to previously published studies [16,17,18,19]. Crude LD_SiAT2 and SL_SiAT2 improved the cell viability of the OMM-1 cell line slightly. Our data indicate that further fractionation could attenuate this effect, as can be seen for the SL fractions in this work, suggesting that this effect may be due to contaminating agents. Glucose, mannitol or guluronic acid were reduced by fractionation. In addition, other not investigated agents like phenols, could have been diminished by purification. Mak et al., 2014 described that crude fucoidans from *Undaria pinnatifida* have a higher toxicity on tumor cells because of the higher yield of uronic acids [27]. However, while the crude fucoidans of this work also have higher amounts of uronic acids compared to the fractions, there were protective for OMM-1. Our fractions have although high molecular weights, which could influence the protective properties [27] and in addition different species may exert different effects.

We also gathered some data indicating that fucoidans can increase the cell viability of ARPE-19 in higher concentrations. It is unknown whether this is due to an actual protective effect of the fucoidan (which is not the case for the oxidative stress protection in ARPE-19, here only 10 µg/mL FE fucoidans had a small protective effect). It could be speculated that fucoidans in higher concentration starting with 100 µg/mL increase the cell metabolism and leads to an increased proliferation rate. It was also reported that 100 µg/mL of commercially available fucoidan from Sigma Aldrich can reduce the apoptosis of ARPE-19 cells via different cellular pathways [28].

### 3.3. Effects on Oxidative Stress Protection-Heterogeneous Results, Dependency on Alginates and Galactose

We also tested the influence of oxidative stress protection of H_2_O_2_ treated OMM-1 and TBHP treated ARPE-19. FE_SAT2ad, FE_SiAT2ad and FE_SiAT3ad at 10 µg/mL showed a small protective effect in ARPE-19, which was also seen in OMM-1 cells. However, the effects of FE was highly heterogeneous as for the FE_SiAT2 extract (no acid precipitation) and also FE_SAT3ad displaying no effect at all. Furthermore, the measured protective effects were small and their biological relevance questionable. This corresponds to early studies, which showed no protective effects of FE fucoidans on oxidative stress toxicity in ARPE-19 [17,19]. As described above, FE_SiAT2 has the highest yield of guluronic and mannonic acid the main component of alginates and lowest mol% of fucose. FE_SAT3ad has the second highest content of guluronic acid. Mannuronic acid yield of FE_SiAT2 is rather high in these samples due to lack of alginate precipitation. It could be speculated that alginates are cumbersome for the protective effects. FE-SAT3ad has the lowest glucose content, an important nutrient for the growth of tumor cells, which could lower the metabolism of cancer cells and interferes with the protective effects on OMM-1 cells. The SL_F2 extract showed the best protective effects in OMM-1 and this is in contrast to the other three SL extracts. The SL_F2 extract had the highest amount of fucose, the main component of fucoidans, with 64.7 mol%. However, SL_F3 had nearly the same content (63.3 mol%), so the loss of the protective effect and overall biological activity against H_2_O_2_ is not due to the fucose content. Also the size and sulfation content between these two SL extracts is similar [15]. It could be suspected that the overall antioxidative effects are due to accompanying substances like phenols [21,29]. The molecular weight and the monosaccharide composition are similar, with the exception that SL_F3 had a higher amount of galactose. It could be speculated that the amount of galactose plays an important role regarding the antioxidative effects, because it is much higher in SL_F3. In relation to heart aging, galactose is described as antioxidants reducing as well as oxidative stress and inflammation inducing [30], so it could interfere with the protective effects. SL_F1 has nearly no fucose but consists of high amounts of alginates. This extract showed no biological activity in any of the tests in this study, which leads us to the conclusion, that algae extracts with lower contents of alginates are recommended for obtaining protective effects.

### 3.4. Effects on VEGF-Acid Precipitation Lowers and Higher Molecular Weight Improves VEGF Inhibition

Several extracts reduced the VEGF secretion after three days of stimulation. FE_SiAT2 was the only FE extract, which inhibited VEGF significantly. This extract has the highest amount of fucose, arabinose/rhamnose and the lowest amount of glucuronic acid, compared to LD_SiAT2 and SL_SiAT2. All other FE extracts were treated with acid and did not lower VEGF significantly. This strongly indicates that acid may alter the structure of FE fucoidan, interfering with VEGF interaction. Indeed, in this study, no fucoidan treated with acid displayed any VEGF reduction effects. The LD extract showed a VEGF inhibiting effect. It had the lowest fucose content out of all extracts, but the highest mannitol content. VEGF inhibition could be caused by mannitol, although this has not yet been investigated no literature regarding the effect of mannitol and VEGF secretion can currently be found. In contrast to the crude SL_SiAT2 and the first fraction SL_F1, the extracts SL_F2 and SL_F3 lowered VEGF very efficiently in comparison to all other extracts. They had both a much higher fucose content and were the purest extracts. The fucose content alone seems not to be the only biological factor contributing to VEGF inhibition, since the FE extracts also had higher fucose content and was not causing VEGF reduction. In addition to fucose content, molecular weight could be of high importance. The two SL fractions SL_F2 and SL_F3 had a significant higher molecular weight (>800 kDa (Table 3)) than all other tested extracts, which supports our findings that bigger fucoidans from *Laminaria hyperborea* were more effective regarding VEGF inhibition [17]. This could be due to a steric interference with the VEGF molecules. This corresponds with previous findings, which suggested that fucoidans with higher molecular weight are generally more anti-angiogenic, while fucoidans with low molecular weight are considered more pro-angiogenic [31].

### 3.5. Comparison of Cellic^®^CTec2 and 3, Alginate Lyases as Well as Precipitation Technique

The Cellic^®^CTec2 vs. Cellic^®^CTec3 relates to different enzyme-mixes added in the first step of the purification procedure. They are both enzyme mixes made for degradation of land plant cell walls. They were applied to degrade the brown algal cell wall cellulose and hemicelluloses as well as the storage compound laminarin. In addition, two different alginate lyases (SigmALy and SALy) were used to degrade the cell wall alginate and together these enzymes release the fucoidans gently from the cell wall matrix. FE extracts of this study can be utilized to compare the biological effects after application of these four enzyme purification techniques. Regarding VEGF inhibition they showed no significant effects with the exception of FE_SiAT2 which was VEGF inhibiting. The use of an additional acid precipitation step seems to be more important for this function than the enzyme mix applied, because only the fraction not treated with acid, FE_SiAT2, exhibited VEGF inhibition. The effects on oxidative stress protection were rather heterogeneous and seem to be more related to the concentration applied. The influence on cell viability of ARPE-19 and OMM-1 were rather similar. Overall, we cannot determine a significant biological difference between the extracts treated with the four enzymatic techniques, suggesting that all methods can be used to purify fucoidans resulting in a slightly different amount of monosaccharide and uronic acid compositions only. Overall, acid precipitation is not recommended, because beneficial biological effects important to AMD could be lost due to a possible removal of sidechains or sulfates from the fucoidans. or perhaps more importantly, reduce the size of the fucoidans by partial hydrolysis of the fucoidan backbone. Since, high molecular weight fucoidans are considered antiangiogenic and of great importance when using fucoidans against AMD [17,31]. CaCl_2_ precipitation of alginate is preferred. Indeed, fucoidans with very high molecular weight of over 800 kDa were obtained in the SL_F2 and F3 fractions treated with CaCl_2_, and these extracts showed the most promising biological effects.

### 3.6. Different Fucoidan Structures between Algal Species Lead to the Described Biological Effects

It is well known that the biological properties of the fuciodans are highly dependent on the fucoidan structure and composition. While no structural data of fucoidan from LD can currently be found in literature, the structure of SL and FE fucoidans is different. FE fucoidan consists of alternating α-(1-3)- and α-(1-4)-linked L-fucopyranose unit with sulfate group primarily at C2 [10,32,33] while sulfate groups have been found at C2 and C4 of some fucose residues [34]. Fucoidans from SL are very diverse. Their structure is depending on the overall harvest time, extraction method and further fractionation, which could lead to mixture of different low-sulfated heteropolysaccharides with proteins and uronic acids [29]. Four partial structures of fucoidans from SL have been reported: fucan sulfate, fucogalactan, fucoglucuronomannan, and fucoglucuronan [29,35]. It can be speculated that the different algal origins with very heterogeneous structural compositions, leads to the different biological effects that we described.

### 3.7. Conclusive Words

This study was well equipped to compare the biological activities of ten fucoidans, with several extract variables. We investigated three different species, from which SL seems most promising. We could demonstrate that the use of acid for alginate precipitation is not recommended and CaCl_2_ should be used. In the four different tested enzymatic treatments, no significant difference could be determined in the biological activities, but the used SAT2 enzyme mix seems very promising considering the activities of the SL_F2 and SL_F3 fractions, that were purified with these enzymes. Also a three-step fractionation after the enzyme treatment with IEX was conducted and we could clearly show that fractionation is recommended to achieve fucoidan extracts with high fucose and low alginate content. The extract SL_F2 which resulted from IEX fractionation after an enzyme assisted extraction with CaCl_2_ precipitation is the most promising extract regarding oxidative stress protection and VEGF inhibition. Considering all tested brown seaweed extracts so far, fractionated SL extracts SL_F2 and SL_F3 are the most efficient regarding VEGF inhibiting effect in ARPE-19. They lowered VEGF nearly to 40%. In previous studies we could determine inhibitory effects of a pure LH fucoidan with nearly 50% at 50 and 100 µg/mL [17] and another SL extract which also lowered the VEGF secretion to nearly 50% at 100 µg/mL [19]. The latter extract contained a very high fucose content of 83.8% and only 6% uronic acids [21] (comparable to SL_F2/F3). Surprisingly, the SL extracts from this study lowered VEGF in all concentrations, already very efficiently at 1 µg/mL. So we can conclude that SL is a promising fucoidan source besides *Laminaria hyperborea* [17] regarding potential treatment of AMD. The enzymatically-assisted extraction method, followed by IEX fractionation seem very promising to obtain highly pure and large sized fucoidans with high content of fucose and low content of alginates. With this novel method fucoidans were produced that showed promising VEGF reducing and anti-oxidative properties. Further research is warranted to confirm the beneficial effects in primary in vitro, ex vivo and in vivo models.

## 4. Material and Methods

### 4.1. Cell Culture

OMM-1, a uveal melanoma cell line [36] was kindly donated by Dr. Sarah Coupland. The cultivation mediumRPMI 1640 (Merck, Darmstadt, Germany) was added with 10% fetal calf serum (Linaris GmbH, Wertheim-Bettingen, Germany) and 1% penicillin/streptomycin (Merck). The human RPE cell line ARPE-19 [37] was bought from ATCC and cultivated in HyClone DMEM (GE Healthcare, München, Germany), supplemented with 1% penicillin/streptomycin, 2.5% HEPES (Merck), 1% non-essential amino acids (Merck) and 10% fetal calf serum. ARPE-19 cells were treated at confluence and OMM-1 cells were treated at subconfluence.

### 4.2. Used Fucoidans, Extraction and Purification Process

#### 4.2.1. Fucoidan Origin

Dried flakes of the brown algae SL were obtained from Icelandic Blue Mussel & Seaweed (Bláskel, Iceland) in June 2017. LD (June 2017) and FE (March 2016) were collected by Coastal Research & Management (Kiel, Germany) in the Kiel Canal. LD and FE seaweeds were washed in demineralized water, lyophilized and grround to a size of approximately 0.25–3 mm.

#### 4.2.2. Alginate Lyase Expression and Purification

The alginate lyase SigmALy was purchased from Sigma-Aldrich (Steinheim, Germany). The alginate lyase SALy A1-II’ from *Sphingomonas* sp. was expressed and purified in *Escherichia coli*, as previously described in Manns et al. [24] with modifications described in Nguyen et al. [15].

#### 4.2.3. Enzyme Assisted Extraction of Brown Seaweed Polysaccharides

The enzymatic extraction of fucoidan was performed in 55 mM phosphate, 15 mM citrate buffer pH 6 with 5% substrate concentration. The enzymes were added with ratio 5% (v/w) for Cellic^®^CTec2/3 (Novozymes A/S, Bagsværd, Denmark), 0.35% (w/w) for alginate lyases. The treatment was performed at 40 °C at 100 rpm. The reaction was stopped by boiling at 90 °C for 10 min and cooling on ice. The supernatant was collected by centrifugation 10 min at 19000 rpm. The fucoidans were precipitated and isolated by addition of 96% EtOH to a final concentration of 72% (except for the SL_F1-F3, where this step was performed after the alginate precipitation). Residual high molecular weight alginates were precipitated either by a two-step acid precipitation (pH 4 followed by pH 2 adjustment with HCl and neutralization with NaOH to pH 7 followed by dialysis in demineralized water using a 3 kDa membrane) or by precipitation with 2% CaCl_2_ (except the LD_SiAT2, FE_SiAT2 and SL_SiAT2 crude extract alginate was not further precipitated). The fucoidans were isolated by centrifugation and lyophilized.

#### 4.2.4. SL Fucoidan Fractionation by Anion-Exchange Chromatography

The SL fucoidans were fractionated as previously described in Nguyen et al. [15]. The fucoidan solution (5 g in 100 mL) was applied to a DEAE-Macroprep column (2.6 cm × 40 cm) in Cl^-^ form. The unbound materials were washed from the column with NaCl 0.1 M and the fucoidans were eluted in concentration gradient of NaCl from 0.1–2 M. The eluates were combined into fractions based on the results of total carbohydrate analysis by phenol-sulfuric acid method [38]. The fractions were passed through a 10 kDa membrane to up-concentrate and remove salt, followed by lyophilization.

Fucoidans were solved in Ampuwa bidest (Fresenius, Schweinfurt, Germany) at concentrations of 1 mg/mL with exception of the three SL fraction SL_F1, SL_F2 and SL_F3 which were soluble at 5 mg/mL. Right before stimulation, used extracts were filtered with 0.2 µm Sarstedt filter (Nümbrecht, Germany) and a dilution series with appropriate medium was performed to get the final concentrations of 1, 10, 50 and 100 µg/mL.

### 4.3. Chemical Composition and Size Distribution Analysis

The monosaccharide composition of the fucoidans was determined as previously described [22]. The size distribution of the fucoidans was determined by HP-SEC as previously described in Nguyen et al. [15] and the pullulan standard was fitted with a Weibull decay model by the following equation: y = a * (1 − EXP(−(x/b) ^ (c))). The sulfate content of the crude extracts were determined as previously described [15] and sulfate content of the SL_F1, F2 and F3 extracts was performed as previously described [15].

### 4.4. Oxidative Stress

#### 4.4.1. OMM-1

Oxidative stress in the uveal melanoma cell line OMM-1 was induced by treatment with 1 mM H_2_O_2_ as previously shown [39]. The appropriate H_2_O_2_ concentration to lower cell viability of OMM-1 cells to nearly 50% after 24 h was previously determined [19] and applied for the assessment of protective effects of fucoidans (1, 10, 50 and 100 µg/mL). Fucoidans were applied 30 min before stress insult. In parallel, cells were treated with the extracted fucoidans only (1, 10, 50 and 100 µg/mL) to determine possible anti-proliferative effects. To measure cell viability, a MTS-assay was performed as described below.

#### 4.4.2. ARPE-19

The appropriate TBHP concentration to lower cell viability of ARPE-19 cells to nearly 50% after 24 h (0.5 mM TBHP) was previously determined [19] and applied for the assessment of protective effects of fucoidans (1, 10, 50 and 100 µg/mL). Fucoidans were applied 30 min before stress insult. In parallel, cells were treated with only the extracted fucoidans (1, 10, 50 and 100 µg/mL) to determine anti-proliferative effects. To measure cell viability the MTS-Assay was performed as described below.

### 4.5. Methyl Thiazolyl Tetrazolium (MTT)-Assay

The established cell viability assay MTT [40] was performed as previously described [13,19] and was applied after three days of stimulation with fucoidans, after collection of the supernatant for VEGF-content assessment. The biological material was washed with PBS and cultivated with 0.5 mg/mL MTT for 2 h. After removal of MTT, cells were treated with dimethyl sulfoxide. Color formation was measured at 550 nm with an Elx800 instrument (BioTek Instruments Inc., Bad Friedrichshall, Germany).

### 4.6. MTS-Assay

For the cell viability and protection assays after 24 h a commercially proliferation assay named “CellTiter 96^®^ AQueous One Solution Cell Proliferation Assay” from Promega Corporation (Mannheim, Germany) was applied as described in the instructions of the supplier. Twenty µL of the MTS solution was added to each treated well of a 96 well plates for 1 h. For the prior treatment of cells, media without phenol red was used.

### 4.7. VEGF ELISA

Secreted VEGF in ARPE-19 supernatant was determined with the Human VEGF DuoSet ELISA Kit from R&D Systems (Wiesbaden, Germany) according to manufacturer’s protocol. Supernatants were collected after three days of stimulation with different fucoidan extracts and an MTT assay was conducted to set the VEGF effect in relation to the cell viability as previously described [19]. The medium was exchanged 24 h before collecting of supernatant.

### 4.8. Statistics

At least four independent experiments per assay have been performed. Statistics have been made with Microsoft Excel (Excel 2010, Microsoft, Redmond, WA, USA) and GraphPad PRISM 7 (GraphPad Software, Inc., San Diego, CA, USA, 2017). One-Way ANOVA was conducted to determine significances. *P* values lower than 0.05 are considered significant. The diagram bars stand for mean and the attached lines for standard deviations.

## 5. Conclusions

Fucoidans or sulfated fucans are very promising marine polysaccharides for a possible new treatment development for AMD. With this study we wanted to test the influence of a new enzymatic purification method on the biological activity of fucoidans from different algal species. We tested ten different fucoidans, from the brown seaweed species LD, SL and FE and compared them with regards to toxicity, oxidative stress protection against H_2_O_2_ or TBHP and VEGF secretion. Furthermore, we assessed which enzymatic purification method (testing five different FE extracts) and how IEX fractionation (testing four different SL extracts) were most promising for these biological activities. No fucoidan displayed a negative effect on viability after 24 h. Some FE fucoidans were slightly (but not relevantly) protective in ARPE-19 cells. SL_F2 and some of the FE extracts showed protective effects in the melanoma cell line OMM-1. Effective extracts reducing VEGF were LD_SiAT2, FE_SiAT2 and SL_F2 and SL_F3 fucoidans. The enzymatic method SAT2 without acid dialysis seems most promising for biological effects, which could be confirmed for with the SL fractions SL_F2 and F3. They were the most effective fucoidans regarding VEGF inhibition, which correlated with a high fucose and low alginate content. SL fucoidans, which were treated with SAT2 enzymes and further processed with ion-exchange chromatography, are the most promising extracts for a potential application in AMD.

## Figures and Tables

**Table 1:** Overview of the used fucoidans, including algae species, extraction method and code used in this manuscript. For the explanation of the extraction methods refer to Section 2.1.

Fucoidan Code	Extraction Method	Algal Species
LD_SiAT2	SigmALY_CTECH2_crude	*Laminaria digitata*
SL_SiAT2	SigmAly_CTECH2_crude	*Saccharina latissima*
FE_SiAT2	SigmAly_CTECH2_crude	*Fucus distichus* subsp. *evanescens*
FE_SiAT2ad	SigmAly_CTECH2_acid_dialysis	*Fucus distichus* subsp. *evanescens*
FE_SiAT3ad	SigmAly_CTECH3_acid_dialysis	*Fucus distichus* subsp. *evanescens*
FE_SAT2ad	Saly_CTECH2_acid_dialysis	*Fucus distichus* subsp. *evanescens*
FE_SAT3ad	Saly_CTECH3_acid_dialysis	*Fucus distichus* subsp. *evanescens*
SL_F1	SALy_CTECH2_CaCl_2__IEX_filtering_Fraction 1 *	*Saccharina latissima*
SL_F2	SALy_CTECH2_CaCl_2__IEX_filtering_Fraction 2 *	*Saccharina latissima*
SL_F3	SALy_CTECH2_CaCl_2__IEX_filtering_Fraction 3 *	*Saccharina latissima*

* Nguyen et al., 2020 [15].

**Table 2:** Overview of monosaccharide and uronic acid composition in mol%. (Fuc-Fucose, Ara/Rham-Arabinose/Rhamnose, Gal-Galactose, Glc-Glucose, Xyl-Xylose, Man-Mannose, GuluA-guluronic acid, GluA-glucoronic acid, ManA-mannonic acid); GuluA + ManA was calculated together and equals mol% alginates; the highest values are marked in bold.

Sample	Fuc	GuluA	ManA	GuluA + ManA	Mannitol	Ara/Rham	Gal	Glc	Xyl	Man	GluA	Total	Sulfate (SO_4_^2−^), %
LD_SiAT2	3.9	12.4	74.8	87.2	**1.2**	0.1	1.4	2.3	1.5	1.2	1.2	100.0	9.3
SL_SiAT2	12.3	**32.2**	48.1	80.3	0.2	0.2	1.3	2.6	0.9	0.8	1.5	100.0	14.4
FE_SiAT2	15.5	18.7	48.8	67.5	0.0	0.3	2.5	2.1	3.6	1.8	6.5	100.0	20.2
FE_SiAT2ad	36.1	7.1	30.0	37.1	0.1	0.6	6.6	**5.7**	**10.2**	1.8	2.0	100.0	30.1
FE_SiAT3ad	35.9	10.0	40.4	50.4	0.1	0.4	2.8	2.2	4.1	2.0	2.2	100.0	29.4
FE_SAT2ad	52.2	8.8	12.5	21.3	0.0	**1.2**	4.8	2.7	9.3	**4.7**	3.9	100.0	31.7
FE_SAT3ad	48.3	11.6	16.4	28.0	0.1	0.7	5.0	2.0	8.3	4.0	3.7	100.0	29.9
SL_F1 *	5.4	8.5	**82.4**	**90.9**	0.0	0.1	0.5	0.4	0.8	0.8	1.1	100.0	6.6
SL_F2 *	**64.7**	0.0	6.9	6.9	0.1	0.3	12.2	0.6	4.8	3.5	**6.9**	100.0	35.6
SL_F3 *	63.3	0.0	0.8	0.8	0.0	0.3	**26.9**	0.4	3.4	2.1	2.8	100.0	**46.4**

* Nguyen et al., [15]. To extract fucoidans in a more gentle way in order to retain the molecule as intact as possible, enzymes were employed in the extraction procedure. Crude fucoidan extracts were prepared using different enzyme cocktails, with either the cell wall degrading enzyme mix Cellic^®^CTec2 or 3 from Novozymes A/S, enzyme cocktails developed to degrade polysaccharides from terrestrial plant cell walls. In addition, alginate lyases were also added, since alginate is a brown seaweed specific polysaccharide that is not degraded by the Cellic^®^CTec2 or 3 [23]. Two different alginate lyases were used (refer Section 4.2.2), which have different specificities [24]. Fucoidans have previously been purified using different enzymes, including the cellulase enzyme Celluclast [25] The Cellic^®^CTec2 used here contains extra β-glucosidases and lytic cellulose monooxygenases (1.14.99.54, 1.14.99.56, AA9) as well as other proprietary proteins compared to Celluclast. In addition, it has specifically been shown that Cellic^®^CTec2 can degrade laminarin [23]. Furthermore, this new method includes the novel use of two different alginate lyases for purification of fucoidans.

**Table 3:** Size and size-distribution of fucoidans determined by HP-SEC.

Fucoidan Code	Fucoidan Size Calculated (kDa)	Fucoidan Size-Distribution Estimated (kDa)
LD_SiAT2	322	250–450
SL_SiAT2	251	100–400
FE_SiAT2	322	100–500
FE_SiAT2ad	366	200–500
FE_SiAT3ad	416	200–500
FE_SAT2ad	366	200–500
FE_SAT3ad	366	200–500
SL_F1 *	Not determined	Not determined
SL_F2 *	>800	100–1000
SL_F3 *	>800	100–1000

* Nguyen et al., 2020 [15].

## Appendix A

**Table A1:** Monosaccharide composition of fucoidans determined by HPAEC, including standard deviations (%mol).

Sample	Fuc	Mannitol	Ara/Rham	Gal	Glc	Xyl	Man	GuluA	GluA	ManA	Total	Sulfate (SO_4_^2−^) mol%
LD_SiAT2	3.9 ± 0.1	1.2 ± 0.1	0.1 ± 0.0	14 ± 0.2	2.3 ± 0.1	1.5 ± 0.1	1.2 ± 0.1	12.4 ± 1.4	1.2 ± 0.1	74.8 ± 1.5	100	9.3 ± 2.4
SL_SiAT2	12.3 ± 0.8	0.2 ± 0.0	0.2 ± 0.0	1.3 ± 1.1	2.6 ± 0.2	0.9 ± 0.0	0.8 ± 0.0	32.2 ± 1.2	1.5 ± 0.2	48.1 ± 0.7	100	14.4 ± 0.6
FE_SiAT2	15.5 ± 0.9	0.0 ± 0.0	0.3 ± 0.0	2.5 ± 0.0	2.1 ± 0.0	3.6 ± 0.1	1.8 ± 0.2	18.7 ± 0.8	6.5 ± 5.5	48.8 ± 3.5	100	20.2 ± 1.5
FE_SiAT2ad	36.1 ± 3.1	0.1 ± 0.1	0.6 ± 0.6	6.6 ± 1.9	5.7 ± 1.5	10.2 ± 3.2	1.8 ± 1.5	7.1 ± 0.7	2.0 ± 0.4	30.0 ± 2.0	100	30.1 ± 0.6
FE_SiAT3ad	35.9 ± 1.2	0.1 ± 0.0	0.4 ± 0.3	2.8 ± 0.7	2.2 ± 0.1	4.1 ± 0.3	2.0 ± 0.1	10. 0 ± 0.2	2.2 ± 0.0	40.4 ± 0.5	100	29.4 ± 1.7
FE_SAT2ad	52.2 ± 1.9	0.0 ± 0.0	1.2 ± 0.2	4.8 ± 0.5	2.7 ± 0.5	9.3 ± 1.1	4.7 ± 0.7	8.8 ± 1.2	3.9 ± 0.3	12.5 ± 0.8	100	31.7 ± 2.0
FE_SAT3ad	48.3 ± 0.6	0.1 ± 0.1	0.7 ± 0.6	5.0 ± 0.7	2.0 ± 0.1	8.3 ± 0.5	4.0 ± 0.7	11.6 ± 0.1	3.7 ± 0.2	16.4 ± 0.3	100	29.9 ± 1.4
SL_F1 *	5.4 ± 1.2	0.0 ± 0.0	0.1 ± 0.0	0.5 ± 0.0	0.4 ± 0.0	0.8 ± 0.1	0.8 ± 0.1	8.5 ± 4.7	1.1 ± 0.1	82.4 ± 4.3	100	6.6 ± 3.6
SL_F2 *	64.7 ± 0.3	0.1 ± 0.0	0.3 ± 0.0	12.2 ± 0.1	0.6 ± 0.1	4.8 ± 0.0	3.5 ± 0.2	0.0 ± 0.0	6.9 ± 0.3	6.9 ± 0.1	100	35.6 ± 2.5
SL_F3 *	63.3 ± 0.7	0.0 ± 0.0	0.3 ± 0.0	26.9 ± 0.3	0.4 ± 0.1	3.4 ± 0.2	2.1 ± 0.1	0.0 ± 0.0	2.8 ± 0.2	0.8 ± 0.1	100	46.4 ± 3.5

* Nguyen et al. 2020 [15].
